# Editorial: A collection of systematic reviews or meta-analyses on the non-pharmaceutical interventions for social, health and psychological well-being

**DOI:** 10.3389/fpsyg.2025.1637687

**Published:** 2025-09-15

**Authors:** Zhenggang Bai, Fang Fu, Iris Chi

**Affiliations:** ^1^Institute of Clinical Research, Evidence-based Medicine Center of Traditional Chinese Medicine, Nanjing University of Chinese Medicine, Nanjing, China; ^2^Department of Social Work, Fudan University, Shanghai, China; ^3^School of Social Work, University of Southern California, Los Angeles, CA, United States; ^4^Sao Center on Aging, University of Hong Kong, Hong Kong, Hong Kong SAR, China

**Keywords:** non-pharmaceutical intervention, evidence based, wellbeing, systematic review, meta-analysis

## Introduction

Evidence-based practice (EBP) seeks to integrate the strongest empirical findings with practitioners' professional judgment while fully respecting the characteristics and preferences of service recipients. Since Sackett et al. first articulated the concept approximately three decades ago (Sackett et al., 1996), it has become the guiding principle in medicine, social work, education, and mental healthcare (Bai et al., 2022). Although randomized controlled trials (RCTs) yield the most rigorous primary data, systematic reviews and meta-analyses prepared in line with PRISMA standards (Moher et al., 2009) and appraised using the GRADE methodology (Guyatt et al., 2011; Higgins et al., 2022) translate scattered results into clear, practice-ready guidance.

The present cross-journal Research Topic (2025), organized with support from the Campbell China Network, assembles five high-quality syntheses that address prevention, treatment, and health promotion across the life course. A concise overview of the contributions is included.

### School-based prevention for adolescents

*Effectiveness of preventive interventions on teens' depression and suicidal tendency* screens 13 RCTs published since 2011 (Ghazal et al.). The review reports modest yet clinically relevant reductions in depressive symptoms when cognitive-behavioral or psycho-educational programs are delivered by mental-health specialists. Owing to considerable heterogeneity in program content, dosage, and follow-up duration, the authors call for future studies to adopt standardized outcome indicators and transparent implementation reports.

### Evolution of dialectical behavior therapy (DBT) research

*Bibliometric analysis of global research on dialectical behavior therapy from 1987 to 2024* examines 2,723 publications and reveals that DBT scholarship has expanded beyond borderline personality disorder into the adolescent, forensic, and digital arenas, yet remains concentrated in the Global North (Shi et al.). The mapping underscores the need for broader international collaboration and culturally responsive adaptations to DBT.

### Culturally informed mind-body intervention

In *research on the intervention effect of Five-Element Music Combined with Eight-Section Brocade on depression among medical students*, 160 students were randomized to music therapy, Qigong exercise, a combined protocol, or usual care (Yao et al.). Both single-component interventions improved depression, anxiety, and sleep, but the combined protocol produced the largest gains, suggesting a synergistic effect that merits wider promotion in campus health services.

### Digital technology for youth mental health

*Promoting mental health in children and adolescents through digital technology* synthesizes 59 studies (10 pooled in meta-analysis) and reports a moderate overall benefit (Hedges g = 0.43) for mobile apps, virtual reality platforms, serious games, and online counseling (Chen et al.). Effect sizes, however, fluctuate with platform type, engagement strategy, and symptom focus, highlighting the importance of detailed process evaluations to elucidate mechanisms of change.

### Exercise and chronic inflammation in healthy populations

*Long-term exercise training and inflammatory biomarkers in healthy subjects* pools 38 RCTs (*n* = 2,557) and confirms that programs lasting at least 12 weeks, delivered at moderate intensity, significantly reduce IL-6, C-reactive protein, and TNF-α (Wang et al.). These findings support exercise as a feasible primary prevention strategy, which may reduce long-term disease risk even in otherwise healthy individuals.

## Emerging themes and implications

Early intervention across multiple domains. The collected studies converge on the value of timely, non-pharmaceutical action, whether via classroom skill-building, digital coaching, culturally grounded mind-body practice, or structured physical activity, in shaping trajectories of wellbeing. This perspective aligns with WHO guidance on adolescent mental-health promotion (World Health Organization, 2020).

Cultural adaptation and contextual relevance. The Five-Element Music plus Eight-Section Brocade trial demonstrates that interventions rooted in indigenous traditions can satisfy strict methodological requirements. At the same time, the DBT bibliometric review reminds us that global applicability cannot be assumed without local evidence.

Methodological progress and remaining challenges. Most reviews comply with PRISMA and register their protocols prospectively, yet gaps persist in the long-term follow-up and reporting of implementation fidelity. Addressing these issues will sharpen future meta-analytic conclusions and enhance knowledge translation.

Interdisciplinary collaboration. The evidence base spans public health, psychology, kinesiology, music therapy, and information technology. Such breadth mirrors the multifaceted nature of real-world problems and calls for integrative intervention packages to be evaluated through collaborative research designs.

## Recommendations for future work

Multi-component trials should examine additive or synergistic effects, as demonstrated in the Five-Element Music plus Eight-Section Brocade study.

Equity-oriented research capacity must be strengthened, especially in low- and middle-income regions, to ensure that global evidence truly represents diverse populations.

Precision implementation science is needed to match interventions to individual and contextual characteristics, thereby realizing the full intent of EBP.

Mechanistic and longitudinal outcomes (e.g., biological markers and digital engagement metrics) should complement symptom measures to clarify how and for whom these interventions work.

## Concluding remarks

This Research Topic underscores that non-pharmaceutical interventions, from school-based cognitive training to traditional mind-body practices, rest on a solid and growing empirical foundation. By conscientiously integrating these high-quality syntheses into routine practice, professionals can deliver person-centered, culturally sensitive, and scientifically informed services that foster social, health, and psychological wellbeing across populations.
